# Host-Filtered Blood Nucleic Acids for Pathogen Detection: Shared Background, Sparse Signal, and Methodological Limits

**DOI:** 10.3390/pathogens15010055

**Published:** 2026-01-06

**Authors:** Zhaoxia Wang, Guangchan Chen, Mei Yang, Saihua Wang, Jiahui Fang, Ce Shi, Yuying Gu, Zhongping Ning

**Affiliations:** Department of Cardiology, Shanghai Pudong New Area Zhoupu Hospital (Shanghai Health Medical College Affiliated Zhoupu Hospital), Shanghai 201318, China; wangzhaoxia_sh@163.com (Z.W.); rocky7233@163.com (G.C.); olive_sh2015@163.com (M.Y.); wangsaihua1004@163.com (S.W.); fangjiahui1980@163.com (J.F.); shice1901@163.com (C.S.)

**Keywords:** plasma cell-free RNA, blood microbiome, host read filtering, low-biomass contamination, tuberculosis, coronary artery disease

## Abstract

Plasma cell-free RNA (cfRNA) metagenomics is increasingly explored for blood-based pathogen detection, but the structure of the shared background “blood microbiome”, the reproducibility of reported signals, and the practical limits of this approach remain unclear. We performed a critical re-analysis and benchmarking (“stress test”) of host-filtered blood RNA sequencing data from two cohorts: a bacteriologically confirmed tuberculosis (TB) cohort (*n* = 51) previously used only to derive host cfRNA signatures, and a coronary artery disease (CAD) cohort (*n* = 16) previously reported to show a CAD-shifted “blood microbiome” enriched for periodontal taxa. Both datasets were processed with a unified pipeline combining stringent human read removal and taxonomic profiling using the latest versions of specialized tools Kraken2 and MetaPhlAn4. Across both cohorts, only a minority of non-host reads were classifiable; under strict host filtering, classified non-host reads comprised 7.3% (5.0–12.0%) in CAD and 21.8% (5.4–31.5%) in TB, still representing only a small fraction of total cfRNA. Classified non-host communities were dominated by recurrent, low-abundance taxa from skin, oral, and environmental lineages, forming a largely shared, low-complexity background in both TB and CAD. Background-derived bacterial signatures showed only modest separation between disease and control groups, with wide intra-group variability. *Mycobacterium tuberculosis*-assigned reads were detectable in many TB-positive samples but accounted for ≤0.001% of total cfRNA and occurred at similar orders of magnitude in a subset of TB-negative samples, precluding robust discrimination. Phylogeny-aware visualization confirmed that visually “enriched” taxa in TB-positive plasma arose mainly from background-associated clades rather than a distinct pathogen-specific cluster. Collectively, these findings provide a quantitative benchmark of the background-dominated regime and practical limits of plasma cfRNA metagenomics for pathogen detection, highlighting that practical performance is constrained more by a shared, low-complexity background and sparse pathogen-derived fragments than by large disease-specific shifts, underscoring the need for transparent host filtering, explicit background modeling, and integration with targeted or orthogonal assays.

## 1. Introduction

Circulating cell-free DNA or RNA (cfDNA or cfRNA) sequencing is increasingly used for non-invasive pathogen detection in blood [1,2,3], yet in low-biomass blood, the non-host signal is often background-dominated and reproducibility remains a concern. After computational removal of human reads, the remaining “non-host” cfRNA can in principle reveal microbial and environmental nucleic acid cfRNA metagenomics (i.e., shotgun host-filtered cfRNA taxonomic profiling) [1,2]. However, the reproducibility of reported signals, and the composition and stability of the background signal, the so-called “blood microbiome”, remain poorly defined, and their impact on pathogen calling is still unclear [4,5].

Multiple studies have reported bacterial DNA in plasma from both patients and healthy individuals, often dominated by taxa classically associated with the oral cavity, skin, or environment [6,7,8]. Whether these signals reflect true low-level translocation, technical contamination, or a mixture of both remains debated [5,8,9]. From a diagnostic perspective, a key practical question is whether the non-host cfRNA fraction contains a relatively stable, low-complexity background that is largely shared across individuals and disease states, thereby compressing the diagnostic dynamic range and constraining the reliable detection of true pathogen-derived fragments [1,4,5].

In parallel, plasma cfRNA metagenomic profiles are highly sensitive to analytical choices. The definition of “non-host” reads depends on the stringency of human read filtering (reference genome, alignment parameters) and on how unmapped reads are extracted (e.g., any-unmapped vs. strictly paired-unmapped reads), and different groups use different taxonomic classifiers such as Kraken [10,11] and MetaPhlAn [12]. Yet there are few systematic benchmarking (“stress test”) comparisons that quantify how these decisions influence the apparent non-host fraction and downstream pathogen calls, making it difficult to distinguish biological signals from pipeline-specific artefacts [8,9,13,14]. The CAD cfRNA cohort (GSE58150) was previously reported to harbor a disease-shifted “blood microbiome” with enrichment of periodontal taxa in CAD cases [15]. The TB plasma cfRNA cohort (GSE255073) was originally analyzed primarily for host cfRNA signatures, without systematic profiling of non-host or pathogen-derived reads [3]. These contrasting prior analyses motivated our unified re-processing of both cohorts to quantify how host-filtering stringency shapes the apparent non-host fraction and downstream taxonomic profiles.

Here, we performed a critical re-analysis and benchmarking (“stress test”) of host-filtered plasma cfRNA datasets from two independent clinical cohorts under a unified pipeline: bacteriologically confirmed tuberculosis (TB; 30 positive vs. 21 negative) and coronary artery disease (CAD; 8 cases vs. 8 controls). We combined stringent human read removal, relaxed (any-unmapped) and strict (paired-unmapped) unmapped-read extraction, and taxonomic profiling with Kraken2 and MetaPhlAn4 with their latest reference databases followed by phylogeny-aware visualization of the resulting microbial communities. Our goals were to quantify (i) how much of the non-host cfRNA fraction can be classified across host-filtering modes and disease groups, (ii) how similar the background community is between an infectious disease (TB) and a non-infectious cardiovascular condition (CAD), and (iii) whether pathogen-derived reads, particularly *Mycobacterium tuberculosis*, emerge as a distinct, diagnostically useful signal beyond the shared background. This work is intended as a benchmarking/limitation study rather than pathogen discovery.

We quantify that only a minority of non-host reads are taxonomically classifiable, with the classified fraction under strict host filtering accounting for a median of 7.3% in CAD and 21.8% in TB (Table 1), showing that this fraction is dominated by recurrent, low-abundance taxa from oral, skin, and environmental lineages, and that the background is highly overlapping between TB and CAD cohorts. *M. tuberculosis* cfRNA is detectable in many TB-positive samples but constitutes ≤ 0.001% of total cfRNA and is observed at similar orders of magnitude in a subset of TB-negative samples, limiting discrimination. These findings support a quantitative benchmark of the background-dominated regime and the practical limits of plasma cfRNA metagenomics for pathogen detection, and they lead us to argue for explicit background modeling, rigorous technical controls, and integration with targeted or orthogonal assays rather than relying on taxonomic abundance differences alone.

Taken together, in this benchmarking/limitation study, we re-analyzed two publicly available plasma cfRNA sequencing cohorts under a unified, version-controlled pipeline, treating host-filtering stringency as an explicit analytical variable. Specifically, we aimed to (i) quantify the classifiable non-host cfRNA fraction under relaxed versus strict host-filtering modes across cohorts and disease groups; (ii) characterize the composition, structure, and cross-cohort overlap of the shared, low-complexity background between an infectious TB cohort and a non-infectious CAD cohort; and (iii) evaluate whether pathogen-derived reads—particularly *M. tuberculosis*—form a distinct, diagnostically useful signal beyond this shared background.

## 2. Materials and Methods

### 2.1. Study Design

**Figure 1:**
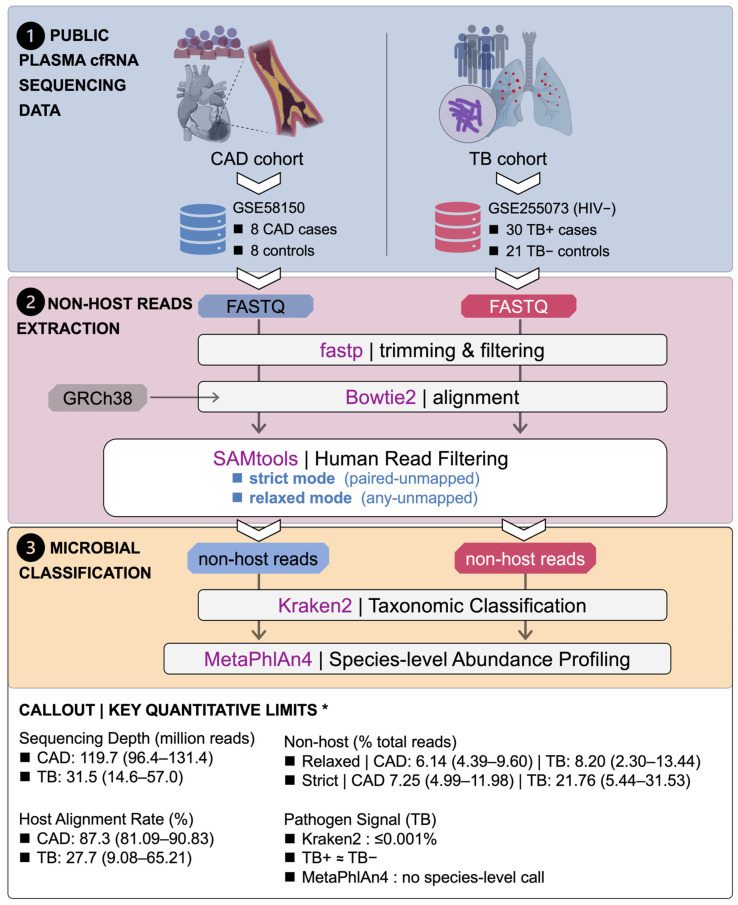
Study design and analysis workflow with key quantitative limits of plasma cfRNA metagenomics. Public plasma cfRNA sequencing datasets from a coronary artery disease (CAD) cohort (GSE58150; 8 CAD cases, 8 controls) and a tuberculosis (TB) cohort (GSE255073; HIV-negative [HIV−] subset, 30 TB-positive [TB+] cases, 21 TB-negative [TB−] controls) were re-analyzed using a unified pipeline. Raw FASTQ files were trimmed and filtered with fastp, aligned to the human genome (GRCh38) with Bowtie2, and subjected to human read filtering with SAMtools to generate two alternative non-host read sets per sample: a strict “paired-unmapped” mode and a relaxed “any-unmapped” mode. Non-host reads from each mode were then used for microbial profiling, with Kraken2 performing taxonomic classification and MetaPhlAn4 generating species-level abundance profiles. A quantitative callout summarizes key dataset constraints (median, range), with values drawn from Table 1 and Supplementary Table S3. Some biological illustrations were created using BioRender (BioRender.com). * TB cohort restricted to the HIV− subset.

The overall study design is summarized in Figure 1. Plasma-derived, cell-free RNA (cfRNA) sequencing datasets from two independent clinical cohorts were re-analyzed using a unified pipeline to assess how human read-filtering stringency and downstream taxonomic profiling influence the apparent “blood microbiome” background and pathogen signal. The tuberculosis (TB) cohort (GSE255073) comprised 51 (30 positive and 21 negative) samples [3], from which all HIV-positive individuals were excluded, and the coronary artery disease (CAD) cohort (GSE58150) comprised 16 samples (8 CAD cases and 8 healthy controls) [16]. Baseline sequencing metrics, including total reads, host alignment rates, and non-host classification rates under relaxed and strict host-filtering modes, are summarized in Table 1, and per-sample metadata and QC metrics are provided in Supplementary Table S1.

### 2.2. cfRNA Sequencing Reads’ Processing and Host Read Filtering

Raw sequencing FASTQ files were retrieved from the NCBI Sequence Read Archive (SRA) using the SRA Toolkit (v3.2.1, https://github.com/ncbi/sra-tools, accessed on 18 March 2025), subjected to adapter trimming and quality filtering with fastp (v1.0.1) [17], then aligned to the human reference genome (GRCh38) using Bowtie2 (v2.5.4). [18] Non-host read pairs with both mates unmapped were extracted with SAMtools (v1.22.1) [19] and used as the starting point for all non-host cfRNA-based taxonomic and microbial profiling analyses (Figure 1).

Human read filtering was formalized under two alternative modes: relaxed (any-unmapped) mode and strict (paired-unmapped) mode. In the relaxed mode, all reads flagged as unmapped in the alignment (SAM flag 0 × 4) were extracted, irrespective of the mapping status of their mate. In the strict mode, only read pairs in which both mates were unmapped to the human reference were retained, and pairs with one mapped and one unmapped mate were discarded. For each sample and for each mode, the fraction of non-host reads was calculated as the number of unmapped reads divided by the total number of sequenced reads and was used to quantify the impact of host-filtering stringency in the TB and CAD cohorts.

### 2.3. Bacterial Taxonomic Classification and Species-Level Abundance Profiling

Non-host cfRNA reads derived from the relaxed and strict host-filtering modes in both cohorts were processed through a unified taxonomic classification and bacterial profiling workflow (Figure 1). Taxonomic classification was performed with Kraken2 (v2.17.1) [10,11] using the standard reference database (k2_standard_08gb_20240605) and default parameters on non-host read pairs from each sample and each host-filtering mode. For each sample, the number of non-host reads classified by Kraken2 and the corresponding classification rate, defined as the proportion of non-host reads receiving a taxonomic assignment, were recorded and compared between relaxed and strict modes within each disease group and between disease and control groups within each cohort. To obtain higher-resolution bacterial community profiles, the same non-host read sets were analyzed with MetaPhlAn4 (v4.2.4) [12] in the paired-end mode using default options and the ChocoPhlAn species genome bin database (mpa_vJan25_CHOCOPhlAnSGB_202503). For each sample, MetaPhlAn4 reported relative abundances for clades from kingdom to species. Downstream analyses focused on bacterial species-level entries, defined as clades with an “s__” prefix. For every detected species, per-sample relative abundances were taken directly from the MetaPhlAn4 output and expressed as percentages. Group-wise mean abundances were then calculated within each cohort (TB-positive vs. TB-negative; CAD vs. control), and species with zero mean abundance in both groups were discarded. For visualization and integration with phylogeny-aware community trees, a total abundance metric was defined for each species as the sum of its mean abundances in the two groups, and a log_10_-transformed value was used to stabilize the dynamic range. Species-level abundance tables and sample-level relative abundance matrices were exported for subsequent statistical analysis. Complete species-level relative abundance matrices for both cohorts are provided in Supplementary Table S2.

### 2.4. Construction of Phylogeny-Aware Community Trees

Phylogeny-aware representations of the non-host bacterial communities were generated by projecting species-level MetaPhlAn4 profiles onto the MetaPhlAn4 reference taxonomy. For each clade reported by MetaPhlAn4, the hierarchical clade string was split into separate taxonomic ranks (kingdom, phylum, class, order, family, genus, and species), with shorter lineages padded by missing values at lower ranks. Directed edges were defined between adjacent ranks (kingdom–phylum, phylum–class, etc.) across all species observed in the TB and CAD MetaPhlAn4 profiles. Nodes were annotated with their taxon names and, for species-level nodes, with group-wise mean relative abundances, total abundance and its log_10_-transformed value, and an indicator of whether the node represented a terminal species (“tip”) or an internal taxonomic level. For each cohort, circular dendrograms were generated using the “dendrogram” layout with the “circular” option enabled. Internal nodes were shown as small gray points and edges as gray links. Species-level nodes were plotted with a point size proportional to the total abundance on a log_10_ scale and fill color encoding their occurrence pattern across clinical groups. In the TB cohort, species were classified as TB-positive only (non-zero in TB-positive and zero in TB-negative), TB-negative only, shared between TB-positive and TB-negative, or other/internal. For the CAD cohort, analogous categories were defined as CAD only, control only, shared between CAD and control, or other/internal. To preserve label readability on the circular layout, labels were restricted to species above a minimal abundance threshold together with a small set of a priori selected key taxa.

### 2.5. Definition of Disease-Associated Taxa and Signature Scores

Based on the species-level MetaPhlAn4 profiles and the phylogeny-aware community trees, a small number of bacterial signatures were pre-defined in each cohort, and sample-level scores were derived by summing the relative abundances of member taxa. In the TB cohort, a four-species background-associated signature (TB_background4) was defined. For each TB sample, the TB_background4 score was obtained by summing the MetaPhlAn4-derived relative abundances (in percentage points) of the four member species. These per-sample scores were then used for group-level summaries and non-parametric comparisons between TB-positive and TB-negative groups. For the CAD cohort, three non-overlapping components were defined. A CAD-enriched signature (CAD_signature) was constructed by aggregating a set of case-biased taxa, a control-enriched signature (CTL_signature) was constructed by aggregating taxa preferentially observed in controls, and a single-species signature was defined to capture the abundance of a dominant skin-commensal taxon. For each CAD sample, CAD_signature and CTL_signature were calculated as the sum of the percentage abundances of their respective member taxa, whereas the single-species signature was taken directly as the percentage abundance of that taxon. These three quantities were used to generate sample-level plots and group-level statistics.

### 2.6. Quantification of M. tuberculosis-Derived Reads

To characterize the mycobacterial signal in the TB cohort, reads assigned to the genus *Mycobacterium* and to the *Mycobacterium tuberculosis* complex (MTBC) were interrogated in both Kraken2 and MetaPhlAn4 outputs. For Kraken2, read counts assigned at or below the *M. tuberculosis* taxonomic node were extracted for each sample and normalized to the total number of cfRNA reads and to the total number of non-host reads. For MetaPhlAn4, species-level relative abundances for any detected MTBC clade/complex-level signal were retrieved. For each of these taxa, mean and median relative abundances were computed separately in TB-positive and TB-negative samples and expressed as percentages of total cfRNA reads. Particular attention was paid to whether these values exceeded or remained below 0.001% of total cfRNA reads.

### 2.7. Statistical Analysis and Data Visualization

All statistical analyses were performed in *R*. Comparisons between host-filtering modes (relaxed versus strict) within the same sample group were carried out using paired Wilcoxon signed-rank tests applied to per-sample percentages of non-host reads or Kraken2-classified reads. Comparisons between disease and control groups within each cohort (CAD versus healthy control; TB-positive versus TB-negative) were conducted using unpaired *Wilcoxon rank-sum* (*Mann–Whitney U*) tests. When multiple species or signatures were evaluated in parallel, *p* values were adjusted using the *Benjamini–Hochberg* procedure to control the false discovery rate, and adjusted *p* values were reported where appropriate. Summary statistics are presented as the mean ± standard deviation, and *p* < 0.05 was considered statistically significant. Figures were generated in *R* using ggplot2 v4.0.1 [20] and ggraph v2.2.2 [21] and further refined in GraphPad Prism (v9.5).

## 3. Results

### 3.1. Host-Filtering Stringency Shapes the Apparent Non-Host cfRNA Fraction More Strongly in TB than in CAD

**Figure 2:**
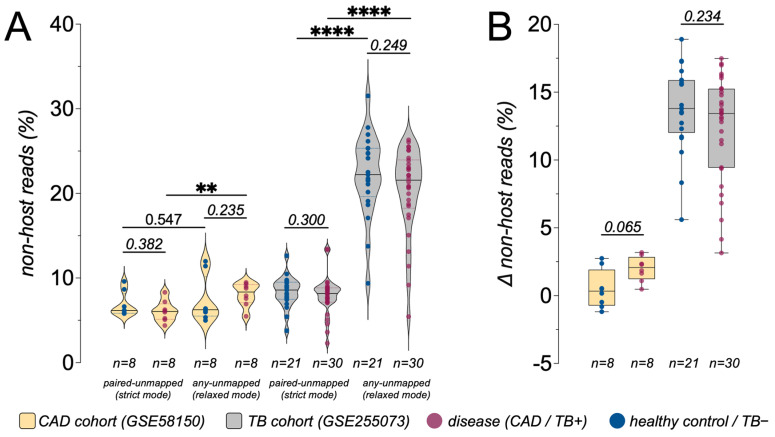
Impact of host-filtering stringency on the proportion of non-host cfRNA reads. (**A**) Violin plots showing the percentage of non-host reads under strict (“paired-unmapped”) and relaxed (“any-unmapped”) human read-filtering modes in the CAD cohort (GSE58150) and TB cohort (GSE255073). Dots represent individual samples (disease, CAD/TB-positive [TB+]; healthy control/TB-negative [TB−]), and dashed lines indicate the median and interquartile range. (**B**) Box-and-whisker plots summarizing the per-sample change in non-host reads (*Δ* non-host reads = strict − relaxed, in percentage points) for each disease group. *p* values above brackets were obtained from unpaired Wilcoxon rank-sum tests comparing *Δ* between CAD controls and CAD cases and between TB− and TB+ samples. The much larger *Δ* in the TB cohort indicates that host-filtering stringency interacts strongly with technical or cohort-specific factors, whereas the CAD cohort shows only small shifts in the apparent non-host fraction. Boxes show the median and interquartile range; whiskers extend to 1.5 × IQR. Statistical significance was determined by appropriate tests as indicated; **** *p* < 0.0001, ** *p* < 0.01. Scatter plots indicate mean ± standard deviation (SD). Sample sizes (n) for each group are shown below the x-axis.

Across both cohorts, only a minority of sequencing reads remained non-host after human read removal and could be classified by Kraken2. Consistent with the different sample types, host alignment rates were high in CAD whole-blood RNA sequencing data (median 87.3%, range 81.09~90.83%) but substantially lower in TB plasma cfRNA data (median 27.7%, range 9.08~65.21%; Table 1 and Table S1). In the CAD cfRNA cohort, the percentage of Kraken2-classified non-host reads was low and relatively tightly distributed under both relaxed (“any unmapped”) and strict (“paired unmapped”) host-filtering modes (Figure 2A). Among the four sample groups (CAD controls, CAD cases, TB-negative, TB-positive), only CAD cases showed a statistically significant difference between relaxed and strict filtering (*p* < 0.01), whereas CAD controls showed only a small, non-significant shift. In contrast, the TB cohort was much more sensitive to host-filtering stringency: both TB-negative and TB-positive groups exhibited markedly higher non-host fractions under the relaxed mode than under the strict mode, with highly significant within-sample differences (*p* < 0.0001; Figure 2A). Under relaxed filtering, a subset of TB samples reached non-host fractions above 20 percent of total reads, yet TB-positive and TB-negative distributions remained largely overlapping, indicating that the dominant effect reflects cohort characteristics or processing choices rather than the TB status itself. Accordingly, all downstream microbial and background analyses were carried out within each cohort, and we did not interpret differences in absolute alignment or classification rates between cohorts as direct biological signals.

This pattern is summarized more directly by examining the per-sample change in classified non-host reads between modes (*Δ* = strict − relaxed; Figure 2B). In the CAD cohort, *Δ* values for both cases and controls were centered near zero and did not differ significantly between groups, indicating only minor effects of filtering stringency. In the TB cohort, *Δ* values had substantially larger magnitudes in both TB-negative and TB-positive groups, reflecting a strong dependence of the apparent non-host fraction on the chosen host-filtering mode, while remaining broadly similar between TB-positive and TB-negative samples.

### 3.2. Plasma Non-Host cfRNA Communities Are Dominated by a Shared, Low-Complexity Background Across Cohorts

**Figure 3:**
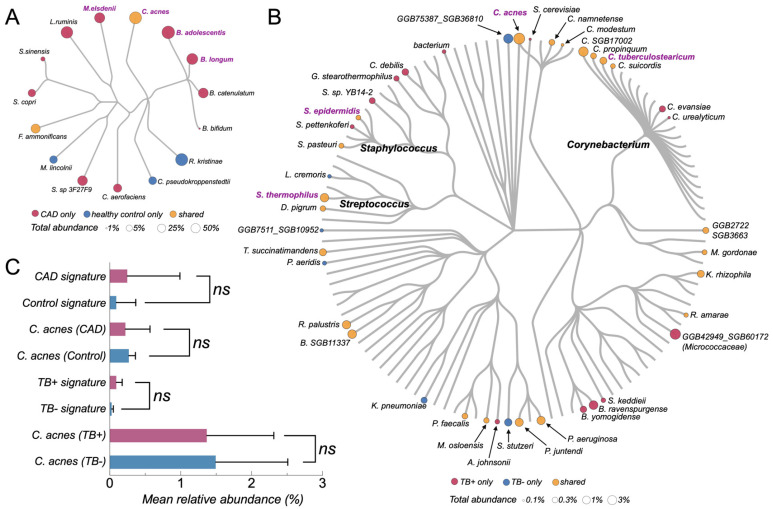
*Cutibacterium acnes*-linked microbial signatures in CAD and TB cfRNA cohorts. (**A**) Microbial phylogram of the CAD cohort (GSE58150) showing taxa detected in plasma cfRNA. Node color encodes occurrence pattern between CAD and control samples (pink, CAD only; blue, control only; orange, shared), and node size reflects total relative abundance across all samples in the cohort. Species labels highlight CAD-enriched (*B. adolescentis*, *B. longum*, *B. catenulatum*, *B. bifidum*, *C. aerofaciens*, *L. ruminis*), control-enriched (*R. kristinae*, *C. pseudokroppenstedtii*), and *C. acnes*. (**B**) Microbial phylogram of the TB cohort (GSE255073) based on non-host cfRNA. Node color indicates occurrence pattern across TB-positive [TB+] and TB-negative [TB−] individuals (pink, TB+ only; blue, TB− only; orange, shared). Node size represents total relative abundance (TB+ plus TB−). Labels mark C. acnes and other highlighted taxa; major clades are annotated at the genus level (*Staphylococcus*, *Streptococcus*, *Corynebacterium*). Circle area proportional to abundance, legend at bottom. (**C**) Composite abundance of cohort-specific signatures and *C. acnes*. Top: CAD cohort. “CAD signature” is the per-sample sum of the CAD-enriched species in (**A**); “Control signature” is the sum of control-enriched species. “C. acnes (CAD)” and “C. acnes (Control)” show *C. acnes* abundance in CAD and control samples, respectively. Bottom: TB cohort. “TB+ signature” and “TB− signature” are the per-sample sums of TB+-only and TB−-only taxa from (**B**). “C. acnes (TB+)” and “C. acnes (TB−)” show C. acnes abundance in TB+ and TB− samples. Bars indicate group means; horizontal whiskers show ±1 SD. “ns” indicates non-significant differences by *Wilcoxon rank-sum* test. X-axis: mean relative abundance (%).

Using the strictly host-filtered non-host cfRNA reads (paired-unmapped mode), we next characterized the composition of bacterial communities in plasma from the TB and CAD cohorts by species-level MetaPhlAn4 profiling and phylogeny-aware visualization (Figure 3).

In the TB cohort, MetaPhlAn4 detected in the order of 10^2^ bacterial species across samples, but the circular community tree showed that a small subset of skin- and environment-associated taxa contributed the majority of the total bacterial signal (Figure 3B). The most prominent nodes included *Cutibacterium acnes*, several *Corynebacterium* species, and coagulase-negative *Staphylococcus*, together forming dense clusters of large, high-abundance tips on the TB tree (Figure 3B, in purple). Many other taxa appeared as isolated, low-abundance terminal nodes scattered across the tree, consistent with a long tail of sporadically detected background species.

In the CAD cohort, the overall non-host cfRNA community was even simpler (Figure 3A). MetaPhlAn4 identified only a few dozen bacterial species across all samples, again dominated by canonical background taxa such as *C. acnes*, *Corynebacterium pseudokroppenstedtii*, *Rothia kristinae*, *Bifidobacterium* spp., and *Collinsella aerofaciens*. The CAD circular tree showed a compact set of high-abundance tips (mainly *Cutibacterium*, *Corynebacterium*, *Rothia*, and *Bifidobacterium* lineages), with relatively few cohort-specific branches unique to CAD cases or controls. Most species either occurred in both disease and control groups or were present only at very low relative abundances.

Comparison of the TB and CAD trees highlighted a substantial overlap in the dominant background lineages across cohorts. High-abundance *C. acnes* and related skin commensals occupied central positions on both trees, and many of the taxa that appeared visually “enriched” in TB-positive plasma belonged to the same broad skin, oral, or environmental clades that were also seen in CAD samples. Conversely, putative pathogen-associated genera contributed only a minute fraction of the total non-host cfRNA signal and did not form a distinct, disease-specific cluster on the phylogenetic backbone. Together, these tree-based views indicate that plasma non-host cfRNA communities in both TB and CAD are dominated by a shared, low-complexity background of recurrent commensal and environmental bacteria, with only modest cohort-specific deviations on top of this common scaffold.

### 3.3. Background-Derived Signature Scores Show Limited Separation Between Disease and Control Groups

As a complementary species-level summary to the phylogeny-aware trees, predefined background-derived signature scores were evaluated in both cohorts (Figure 3C). These scores aggregated relative abundances of recurrent taxa into three components: a CAD-enriched signature, a control-enriched signature, and the single-species abundance of *Cutibacterium acnes.* In the CAD cohort, the CAD-enriched signature (sum of *Bifidobacterium* spp. together with *Collinsella*/*Ligilactobacillus taxa*) showed higher median values in CAD cases than in controls, but with substantial overlap between groups and several CAD samples close to zero. The control-enriched signature was largely confined to controls, with most CAD cases showing negligible values. In contrast, the relative abundance of *C. acnes* was low and broadly similar in CAD and control plasma cfRNA, without a clear group-level shift. In the TB cohort, four frequent background taxa—*C. acnes*, *Corynebacterium tuberculostearicum*, *Staphylococcus epidermidis*, and *Saccharomyces cerevisiae*—were present at comparable orders of magnitude in both TB-positive and TB-negative samples. Their sample-level distributions were wide within each group and strongly overlapping between groups, and no single species showed a consistent increase or decrease restricted to TB-positive and TB-negative plasma. Taken together, these background-derived signatures show only modest separation between disease and control groups and are dominated by intra-group variability, consistent with a shared, low-complexity background community rather than a disease-specific cfRNA microbiome pattern.

In the TB cohort, reads assigned to the MTBC were extremely scarce when re-examined in both Kraken2 and MetaPhlAn4 outputs (Supplementary Table S3). Supplementary Table S3 summarizes TB-positive versus TB-negative medians and ranges for MTBC-assigned reads (absolute and relative) and reports detection concordance between the two classifiers. Across TB-positive and TB-negative samples, the *Mycobacterium*-derived signal typically remained at or below ~0.001% of total plasma cfRNA reads, with overlapping ranges between groups and frequent detection of low-level non-tuberculous species such as *M. gordonae* and *M. paragordonae*. As a result, mycobacterial cfRNA abundance in plasma did not provide robust separation between TB-positive and TB-negative individuals and was therefore not pursued further as a standalone discriminatory marker.

## 4. Discussion

### 4.1. Host-Filtering Stringency Has an Asymmetric Impact on Apparent Non-Host cfRNA Between TB and CAD

We re-analyzed publicly available tuberculosis (TB) and coronary artery disease (CAD) cfRNA datasets using a unified pipeline, treating host-filtering stringency as an explicit analytical variable (Figure 1). Comparing relaxed (“any-unmapped”) versus strict (“paired-unmapped”) filtering on identical raw data showed that host depletion definitions alone substantially reshape the apparent non-host fraction entering taxonomic profiling (Figure 2A,B). The impact was strongly cohort-asymmetric: the TB cohort showed large shifts between modes in both TB-positive and TB-negative samples, whereas CAD exhibited only modest changes, with significant differences confined to CAD cases (Figure 2A). This pattern aligns with cohort-level mapping/host depletion differences (Table 1 and Table S1) and is reflected by much larger per-sample *Δ* non-host values in TB than in CAD (Figure 2B).

These results demonstrate that the “non-host fraction” is operational rather than intrinsic, hinging on whether orphan mates and discordant pairs are retained. Therefore, generic statements such as “human reads were removed and unmapped reads were analyzed” are insufficient for reproducibility. In low-biomass cfRNA pathogen studies, host-filtering parameters should be explicitly reported and sensitivity to relaxed versus strict definitions should be evaluated to reduce over-interpretation of background-dominated signals [5,8].

### 4.2. Shared Low-Complexity Background Constrains Disease Discrimination in Plasma cfRNA

Across TB and CAD cohorts, non-host cfRNA communities were dominated by a small, recurrent set of skin-, oral-, and environmental-associated taxa. MetaPhlAn4 consistently identified genera such as *Cutibacterium*, *Corynebacterium*, and *Staphylococcus* as major contributors to the classified fraction, recurring across samples and host-filtering modes at comparable orders of magnitude in cases and controls (Figure 3A,B). Phylogeny-aware community trees showed highly overlapping structures between cohorts, with no clear partition into “TB-specific” versus “CAD-specific” communities (Figure 3A,B), supporting a reproducible, low-complexity background embedded within an overwhelmingly host-derived library [5,8,13,14]. A full list of detected species and per-sample relative abundances is provided in Supplementary Table S2.

We next tested whether background-associated taxa could separate disease from controls. In CAD, CAD-enriched and control-enriched composite signatures showed the expected directionality but remained highly overlapping, with wide inter-sample variability; the dominant background species C. acnes also did not differ significantly between CAD cases and controls (Figure 3C). These observations are consistent with prior reports of detectable bacterial nucleic acids in CAD blood while indicating modest effects that do not yield robust classifiers [15]. In TB, common background taxa similarly occurred at overlapping orders of magnitude in TB-positive and TB-negative samples without consistent separation (Figure 3C). Collectively, these results indicate a limited discriminative value of background-derived abundance signatures in plasma cfRNA and highlight the risk of over-interpreting small fold-changes in ubiquitous taxa without background-aware modeling, appropriate controls, and external validation.

Notably, because the original studies lacked extraction blanks and library negative controls, we use the term ‘background’ in an intentionally agnostic sense and cannot disentangle low-level biological translocation from technical contamination (including reagent- and laboratory-introduced taxa) in these public, de-identified datasets. Consequently, the absence of negative controls limits our ability to attribute low-abundance taxa to true biology rather than reagents or other technical sources, and such signals are not reliably interpretable without appropriate negative controls and orthogonal validation. Finally, phylogeny-aware overlap/consistency analyses can describe a shared community structure, but they cannot substitute for true negative controls.

### 4.3. Mycobacterial cfRNA Signal Is Extremely Low and Overlaps Between TB-Positive and TB-Negative Plasma

Because the TB cohort targets tuberculosis diagnosis, we examined *Mycobacterium*-derived cfRNA using Kraken2 assignments to the genus *Mycobacterium* and the MTBC, together with MetaPhlAn4 species profiles (Supplementary Table S3). Although *Mycobacterium*-assigned reads were detectable in many TB-positive samples, they represented only an ultra-low fraction of the reads processed after host filtering (i.e., the non-host reads input to taxonomic profiling) and remained a small subset of the already limited non-host component (Supplementary Table S3, which summarizes TB-positive vs. TB-negative medians and ranges for absolute counts and relative metrics). Signals from non-tuberculous lineages (e.g., *M. gordonae*) were sometimes comparable to or higher than MTBC, and a low-level *Mycobacterium* signal also appeared in a subset of TB-negative samples at overlapping orders of magnitude. Notably, MetaPhlAn4 did not report a species-level *M. tuberculosis* signal in these samples, resulting in no cross-tool concordant positives at the species level (Supplementary Table S3), thus highlighting classifier dependence in the ultra-low-abundance regime. Consistent with the extreme sparsity of pathogen cfRNA in plasma without microbial enrichment [3], this regime blurs the true pathogen signal versus cross-mapping, contamination, and the stochastic background, making low-count Mycobacterium reads unreliable for TB-positive versus TB-negative discrimination. Robust plasma TB detection will therefore require targeted enrichment and optimized library preparation plus explicit background modeling and orthogonal evidence, rather than unsupervised taxonomic profiles alone.

### 4.4. Methodological, Reporting, and Translational Implications for Blood-Based Metagenomic Pathogen Detection

Beyond the biological findings, this re-analysis highlights practical constraints that currently limit plasma cfRNA/cfDNA metagenomic pathogen detection and can inflate apparent “blood microbiome” signals. Both cohorts were reprocessed under a fully specified, up-to-date, unified pipeline (Figure 1), using *fastp* v1.0.1 for adapter/quality trimming [17,22] and *Bowtie2* v2.5.4 (GRCh38) for stringent host depletion rather than splice-aware RNA aligners (e.g., *HISAT2*) [18,23]. Critically, host filtering was treated as an explicit analytical variable: relaxed “any-unmapped” versus strict “paired-unmapped” definitions shifted the apparent non-host fraction by amounts comparable to, or larger than, disease–control differences (Figure 2A,B), demonstrating that undocumented unmapped-read definitions undermine reproducibility in low-biomass settings [5,8]. The CAD cohort further serves as a reproducibility stress test: previously reported disease-shifted profiles based on *Kraken* v1 and earlier references [15] were not reproduced when re-analyzed with Kraken2 (v2.17.1; 2024 standard database) [10,11] and MetaPhlAn4 (v4.2.4; expanded marker set including 26,970 species-level genome bins with MAG-derived candidates) [12], instead recapitulating the same low-complexity background structure described above (Figure 3).

These results motivate a minimal set of reporting and design requirements for future blood-based metagenomic pathogen studies: (i) explicitly specify host depletion parameters (aligner, reference build, MAPQ/pairedness rules, multi-mapping handling) and report per-sample host/non-host read statistics (e.g., Table S1); (ii) plan sequencing depth around realistic classifiable non-host fractions and sparse pathogen signal, rather than assuming deeper sequencing will reveal a rich “blood microbiome”; (iii) incorporate and report negative controls (extraction blanks and library controls), and treat recurrent low-abundance taxa across samples/controls as background candidates unless supported by orthogonal evidence; and (iv) adopt conservative species-level pathogen criteria that integrate read count/abundance thresholds, cross-sample consistency, and concordance with microbiologic or clinical data, given that ultra-sparse reads can overlap between disease-positive and disease-negative samples (Figure 3).

Finally, the observed regime—dominant shared background with ultra-scarce pathogen fragments—suggests that improved diagnostics will likely require biochemical enrichment and orthogonal layers rather than incremental computational filtering alone. Antibody-based enrichment has demonstrated a high sensitivity in low-input settings (cfMeDIP-seq) [24,25,26], and prokaryote-skewed nucleic acid features—particularly adenine methylation-associated signals linked to restriction modification and virulence regulation [27,28,29], contrasted with the lack of reproducible genomic adenine methylation in healthy human tissues [30]—highlight a plausible enrichment axis for pathogen-informative fragments. Together with modification-aware single-molecule sequencing approaches [31], these observations motivate integrated assay designs that combine stringent, transparent host filtering and explicit background modeling with selective capture and orthogonal validation, using the quantitative baseline established here (Figure 1, Figure 2 and Figure 3) as a benchmark.

### 4.5. Limitations

This study is a secondary re-analysis of publicly available, de-identified datasets and is therefore constrained by the experimental design and metadata of the original studies. Most importantly, neither cohort included extraction blanks or library negative controls, which limits our ability to distinguish true low-level biological signal from reagent- or laboratory-introduced contamination and to confidently interpret low-abundance taxa. In this context, phylogeny-aware overlap/consistency analyses can characterize a shared community structure but cannot substitute for true negative controls. Statistical inference is limited by the sample size (particularly the CAD cohort, eight cases vs. eight controls) and by the sparsity of classifiable non-host reads; modest disease-associated shifts may therefore be underpowered, and weak or null differences should not be over-interpreted as evidence of absence. Finally, because no new wet-lab validation can be performed within a re-analysis framework, pathogen-associated findings should be interpreted cautiously and ideally confirmed using orthogonal assays in prospectively controlled studies.

## Figures and Tables

**Table 1:** Summary of tuberculosis and coronary artery disease cfRNA cohorts and sequencing metrics.

Cohort	CAD (GSE58150)	TB * (GSE255073)
Sample type	whole blood RNA	plasma cfRNA
Size (*n*)	16	51
Disease cases/controls, (*n*)	8/8	30/21
Total reads (million)	119.7 (96.4–131.4)	31.5 (14.6–57.0)
Host alignment rate (%)	87.3 (81.09–90.83)	27.7 (9.08–65.21)
non-host reads, relaxed (%)	6.14 (4.39–9.60)	8.20 (2.30–13.44)
non-host reads, strict (%)	7.25 (4.99–11.98)	21.76 (5.44–31.53)

Values are reported as median (range). “Non-host reads” indicate the percentage of total reads assigned by Kraken2 to non-host taxa under the indicated host-filtering modes. CAD, coronary artery disease; TB, tuberculosis. * For the TB cohort, counts and summary statistics are restricted to HIV-negative participants; HIV-positive samples (*n* = 10) were excluded from microbiome analyses.

## Data Availability

Raw cfRNA-seq data from publicly available GEO datasets (GSE58150 and GSE255073) were downloaded from the NCBI Gene Expression Omnibus and reprocessed as described in the Section 2. All intermediate data matrices and analysis scripts generated in this study have been deposited in a public repository (https://github.com/nzp-pvg/cfRNA-Microbiome, accessed on 1 December 2025) and are freely available under the MIT license. The repository includes per-sample Kraken2 reports and MetaPhlAn4 profiles, along with the summary tables/matrices used for downstream analyses and figure generation. Processed non-host FASTQ files are not redistributed; instead, they can be reproducibly regenerated from the original public accessions using the provided workflow script (01_download_raw_fastq.sh and 02_host_mapping_and_nonhost_extraction.sh).

## Supplementary Materials

The following supporting information can be downloaded at: https://www.mdpi.com/article/10.3390/pathogens15010055/s1, Table: S1. Sample-level sequencing, host alignment, and Kraken2 classification metrics (uploaded as a separate Excel); Table S2. Per-sample taxonomic profiles and relative abundances from Kraken2 and MetaPhlAn4 (uploaded as a separate Excel file); Table S3. Sample-level and group-level MTBC signal in the TB cohort (Kraken2 vs. MetaPhlAn4) (uploaded as a separate Excel file).

## Abbreviations

cfDNA, cell-free DNA; cfRNA, cell-free RNA; TB, tuberculosis; CAD, coronary artery disease; TB+, TB-positive; TB−, TB-negative; HIV−, HIV-negative; SRA, Sequence Read Archive; IP, immunoprecipitation; cfMeDIP-seq, cell-free methylated DNA immunoprecipitation sequencing; GEO, Gene Expression Omnibus; IQR, interquartile range; Kraken2, *Kraken* version 2; MetaPhlAn4, MetaPhlAn version 4; NCBI, National Center for Biotechnology Information; RNA-seq, RNA sequencing; SD, standard deviation; *Δ*, difference (as defined in the corresponding figure/analysis); MTBC, Mycobacterium tuberculosis complex; *C. acnes*, *Cutibacterium acnes*; *M. elsdenii*, *Megasphaera elsdenii*; *B. adolescentis*, *Bifidobacterium adolescentis*; *B. longum*, *Bifidobacterium longum*; *B. catenulatum*, *Bifidobacterium catenulatum*; *B. bifidum*, *Bifidobacterium bifidum*; *C. aerofaciens*, *Collinsella aerofaciens*; *L. ruminis*, *Ligilactobacillus ruminis*; *R. kristinae*, *Rothia kristinae*; *C. pseudokroppenstedtii*, *Corynebacterium pseudokroppenstedtii*; *F. ammonificans*, *Faecalibacterium ammonificans*; *M. lincolnii*, *Moraxella lincolnii*; *S.* sp. *3F27F9*, *Sphingomonas* sp. *3F27F9*; *S. sinensis*, *Segatella sinensis*; *S. copri*, *Segatella copri*; *S. epidermidis*, *Staphylococcus epidermidis*; *S. thermophilus*, *Streptococcus thermophilus*; *C. tuberculostearicum*, *Corynebacterium tuberculostearicum*; *S. cerevisiae*, *Saccharomyces cerevisiae*; *K. pneumoniae*, *Klebsiella pneumoniae*; *P. faecalis*, *Pseudomonas faecalis*; *M. osloensis*, *Moraxella osloensis*; *S. stutzeri*, *Stutzerimonas stutzeri*; *A. johnsonii*, *Acinetobacter johnsonii*; *P. aeruginosa*, *Pseudomonas aeruginosa*; *P. juntendi*, *Pseudomonas juntendi*; *S. keddieii*, *Sanguibacter keddieii*; *B. ravenspurgense*, *Brevibacterium ravenspurgense*; *B. yomogidense*, *Brevibacterium yomogidense*; plus unnamed metagenome-assembled genomes *GGB75387_SGB36810*, *GGB2722_SGB3663*, and *GGB42949_SGB60172* (family *Micrococcaceae*).
